# Pancreatic fibroblast growth factor 21 protects against type 2 diabetes in mice by promoting insulin expression and secretion in a PI3K/Akt signaling‐dependent manner

**DOI:** 10.1111/jcmm.14007

**Published:** 2018-11-20

**Authors:** Yingying Pan, Baile Wang, Jujia Zheng, Rongrong Xiong, Zhichao Fan, Yanna Ye, Saisai Zhang, Qinyao Li, Fanghua Gong, Chaoming Wu, Zhuofeng Lin, Xiaokun Li, Xuebo Pan

**Affiliations:** ^1^ School of Pharmaceutical Sciences Wenzhou Medical University Wenzhou China; ^2^ State Key Laboratory of Pharmaceutical Biotechnology Department of Medicine The University of Hong Kong Hong Kong; ^3^ The Second Affiliated Hospital of Wenzhou Medical University Wenzhou China; ^4^ The First Affiliated Hospital of Jinan University Guangzhou China

**Keywords:** diabetes, FGF21, insulin, pancreatic β‐cell

## Abstract

Fibroblast growth factor 21 (FGF21) is important in glucose, lipid homeostasis and insulin sensitivity. However, it remains unknown whether FGF21 is involved in insulin expression and secretion that are dysregulated in type 2 diabetes mellitus (T2DM). In this study, we found that FGF21 was down‐regulated in pancreatic islets of db/db mice, a mouse model of T2DM, along with decreased insulin expression, suggesting the possible involvement of FGF21 in maintaining insulin homeostasis and islet β‐cell function. Importantly, FGF21 knockout exacerbated palmitate‐induced islet β‐cell failure and suppression of glucose‐stimulated insulin secretion (GSIS). Pancreatic FGF21 overexpression significantly increased insulin expression, enhanced GSIS, improved islet morphology and reduced β‐cell apoptosis in db/db mice. Mechanistically, FGF21 promoted expression of insulin gene transcription factors and soluble N‐ethylmaleimide‐sensitive factor attachment protein receptor (SNARE) proteins, the major regulators of insulin secretion, as well as activating phosphatidylinositol 3‐kinase (PI3K)/Akt signaling in islets of db/db mice. In addition, pharmaceutical inhibition of PI3K/Akt signaling effectively suppressed FGF21‐induced expression of insulin gene transcription factors and SNARE proteins, suggesting an essential role of PI3K/Akt signaling in FGF21‐induced insulin expression and secretion. Taken together, our results demonstrate a protective role of pancreatic FGF21 in T2DM mice through inducing PI3K/Akt signaling‐dependent insulin expression and secretion.

## 1 INTRODUCTION

Type 2 diabetes mellitus (T2DM) is the predominant form of diabetes and is characterized by insulin resistance and pancreatic β‐cell failure, which lead to abnormally high blood glucose levels (hyperglycaemia).^1^, ^2^ Impaired function of β‐cells, which are responsible for the production and secretion of insulin, is a critical contributing factor for the progression from prediabetes to diabetes.^3^, ^4^ The loss of responding β‐cells in pancreatic islets has been found in genetically diabetic (db/db) mice.^5^ Although previous studies have reported that the β‐cell‐specific transcription factors pancreatic duodenal homeobox 1 (PDX‐1) and v‐Maf musculoaponeurotic fibrosarcoma oncogene family, protein A (MafA) play key roles in the maintenance of β‐cell function and the production of insulin,^6^, ^7^ the precise molecular mechanisms underlying β‐cell function and insulin expression remain largely unknown, thus greatly limiting the development of effective therapeutic strategies against T2DM.

Fibroblast growth factor 21 (FGF21) is a member of the FGF subfamily but lacks mitogenic activity.^8^ Increasing evidence has shown that FGF21 is overexpressed in response to fasting/starvation and mediates fatty acid metabolism, ketogenesis and growth hormone resistance.^9^, ^10^, ^11^ In addition, administration or ectopic overexpression of FGF21 protects against obesity and obesity‐associated metabolic disorders in both rodent and nonhuman primate models.^12^, ^13^, ^14^ Mechanistically, FGF21 functions through activating FGF receptor 1/β‐klotho complex, which is abundant in adipose tissue as well as the liver, pancreas and hypothalamus.^12^, ^15^, ^16^ FGF21 has been shown to play important roles in cellular processes in adipocytes, including glucose uptake, lipolysis,^17^ mitochondrial fatty acid oxidation,^18^ peroxisome proliferator‐activated receptor‐γ activation^19^ and white adipose browning.^20^

On the other hand, FGF21 also has significant effects on hepatic glucose homeostasis and hepatic insulin sensitivity in T2DM mice.^12^ Moreover, FGF21 has a high basal expression in pancreatic islets of mice^21^, ^22^, ^23^ as well as enhances β‐cell function and survival in diabetic mice.^24^, ^25^ Importantly, exogenous FGF21 replenishment has been shown to increase insulin secretion and content in pancreatic islets isolated from diabetic rodents.^25^ However, the role of pancreatic FGF21 in the maintenance of pancreatic islet morphology and function remains obscure. In this study, we investigated the expression pattern and role of FGF21 in pancreatic islets using a T2DM mouse model and proposed the underlying regulatory mechanism.

## 2 MATERIALS AND METHODS

### 2.1 Animal study

FGF21‐knockout mice using the C57BL/6J genetic background were generated as described previously.^26^ Ten‐week‐old male T2DM BKS.Cg‐Dock7^m^+/+Lepr^db^/J mice (BKS‐db/db mice) and lean controls were purchased from The Jackson Laboratory (#000642; Bar Harbor, ME, USA). The mice were housed in clean cages with a regular 12‐hour dark/light cycle and had free access to food and water. The animal experiments were carried out in accordance with Wenzhou Medical University Guidelines for the Care and Use of Laboratory Animals (wydw2015‐0096). Adeno‐associated viral (AAV, serotype 5) vector encoding green fluorescent protein (GFP) driven by the modified mouse insulin promoter and the AAV helper vector were purchased from Viraltherapy Technologies (Viraltherapy Technologies, Hubei, China). To determine the effect of FGF21 on insulin synthesis and secretion in β‐cells, 16‐week‐old db/db mice were treated with AAV‐GFP or AAV‐FG F21 carrying the mouse insulin promoter by tail intravenous injection at a final dose of 1 × 10^11^ vg/mouse in a total volume of 100 μL. The intraperitoneal glucose tolerance test was performed at 4 weeks after the treatment. Mice were fasted overnight (17:00 to 9:00) prior to the intraperitoneal injection of 1.0 g/kg body weight of glucose. Blood glucose levels were measured using a glucometer (B. Braun, Germany) loaded with a small drop of blood (~5 μL) from the tail tip at 15, 30, 45, 60, 75, 90 and 120 minutes after the administration of glucose. After the intraperitoneal glucose tolerance test, the animals were killed and the following experiments were performed.

### 2.2 Pancreatic islet isolation and primary culture

The animals were dissected, and the pancreatic tissue was exposed, followed by perfusion with 2 mL of type IV collagenase (2 mg/mL; Sigma) via the bile duct. The tissue was then digested by shaking gently for 10 minutes at 37°C. Digestion was terminated by perfusion with 30 mL of Hank's solution, and then the islets were hand‐picked under a stereo microscope and maintained in RPMI‐1640 culture medium (Gibco, Life Technologies) supplemented with 10% foetal bovine serum (Gibco, Life Technologies) and 100 U/mL penicillin–0.1 mg/mL streptomycin (Invitrogen, VIC, Australia) overnight. The isolated islets were washed twice and pre‐incubated in Krebs‐Ringer bicarbonate buffer containing 0.1% fatty acid‐free bovine serum albumin and 3 mmol/L glucose for 1 hour, followed by stimulation with 16.7 mmol/L glucose for 1 hour. Insulin secretion in each fraction was measured using an insulin enzyme‐linked immunosorbent assay (ELISA) kit (Antibody and Immunoassay Services, The University of Hong Kong). For signaling studies, the freshly isolated islets were serum‐starved for 8 hours and then treated with 1 mmol/L palmitate for 48 hours.

### 2.3 Cell viability assay

Islet viability was detected by fluorescein diacetate/propidium iodide staining. Fluorescein diacetate (Sigma Aldrich, St. Louis, MO, USA) stains living cells, exhibiting green fluorescence; while propidium iodide (Sigma Aldrich) stains dead cells, exhibiting red fluorescence. Islets were incubated with fluorescein diacetate for 2 minutes, followed by incubation with propidium iodide for 30 seconds. Then, the islets were washed three times with phosphate‐buffered saline and observed under a fluorescence microscope (Nikon Eclipse TE 200‐S, Chiyoda‐Ku, Japan) at 200× magnification.

### 2.4 Immunofluorescence and immunohistochemistry

Mouse pancreatic tissues were collected, fixed in paraformaldehyde, dehydrated and then embedded in paraffin. Five‐micrometer thick sections were prepared for immunofluorescence staining. Briefly, after deparaffinization and rehydration, sections were blocked in 5% bovine serum albumin, followed by incubation with primary antibodies overnight at 4°C, and then with FITC‐ or TRITC‐conjugated secondary antibodies (Santa Cruz) for 60 minutes at 37°C. The sections were washed with phosphate‐buffered saline–Tween 20 three times, then mounted with DAPI (Abcam) for nuclear staining. For immunohistochemical staining, horseradish peroxidase‐conjugated anti‐rabbit secondary antibodies were used, and the staining was visualized using diaminobenzidine, followed by counterstaining with haematoxylin. The following primary antibodies were used: anti‐amylase (sc‐46657; Santa Cruz), anti‐insulin (sc‐9168; Santa Cruz), anti‐insulin (ab6995; Abcam), anti‐glucagon (sc‐13091; Santa Cruz), anti‐FGF21 (ab64857; Abcam), anti‐syntaxin‐1 (STX‐1) (sc‐12736; Santa Cruz), anti‐SNAP25 (ab41455; Abcam), anti‐VAMP2 (ab3347; Abcam) and anti‐cleaved caspase‐3 (9664; Cell Signaling Technology).

### 2.5 Biochemical measurements

Blood glucose levels were measured using a standard automated glucose monitor (B. Braun, Germany). Plasma insulin levels were determined using a high‐sensitive mouse‐insulin ELISA Kit (Antibody and Immunoassay Services, The University of Hong Kong). FGF21 levels were measured using a mouse‐FGF21 ELISA Kit (Antibody and Immunoassay Services, The University of Hong Kong).

### 2.6 Western blot

Briefly, tissues or islets were homogenized and lysed with RIPA buffer supplemented with a Complete Mini Protease Inhibitor Cocktail (Roche, Basel, Switzerland) and phosphatase inhibitors. Lysates were obtained, and protein concentrations were determined using a bicinchoninic acid protein assay. Protein samples (20 μg) were loaded, separated by sodium dodecyl sulfate–polyacrylamide gel electrophoresis and transferred onto a nitrocellulose membrane. The membrane was blocked with 5% non‐fat dry milk in Tris‐buffered saline–Tween 20, then incubated with primary antibodies overnight at 4°C and finally incubated with horseradish peroxidase‐conjugated secondary antibody for 1 hour at 37°C. Detection was performed with enhanced chemiluminescence reagents (Advansta). The antibodies used were as follows: anti‐MafA (sc‐66958; Santa Cruz), anti‐MafB (sc‐10022; Santa Cruz), anti‐PDX‐1 (sc‐14662; Santa Cruz), anti‐STX‐1 (sc‐12736; Santa Cruz), anti‐FGF21 (ab171941; Abcam), anti‐SNAP25 (ab41455; Abcam), anti‐VAMP2 (ab3347; Abcam), anti‐cleaved caspase‐3 (9664; Cell Signaling Technology), anti‐caspase‐3 (9662; Cell Signaling Technology), anti‐phospho‐phosphatidylinositol 3‐kinase (PI3K) at Tyr458/Tyr199 (4228; Cell Signaling Technology), anti‐total PI3K (4249; Cell Signaling Technology), anti‐phospho‐Akt at Ser‐473 (4060; Cell Signaling Technology), anti‐total Akt (4685; Cell Signaling Technology) and anti‐GAPDH (AB‐P‐R001; GoodHere Technology).

### 2.7 Real‐time PCR analysis

Total RNA was extracted from tissue or isolated islets using TRIzol reagent (Invitrogen). cDNA was synthesized from 0.5 μg of total RNA using a supermix consisting of oligo (dT), random hexamer primers and reverse transcriptase (Invitrogen). Relative gene expression levels were determined by reverse transcription–polymerase chain reaction (PCR) (Applied Biosystems, Foster City, CA, USA) using SYBR Green with normalization to GAPDH. The primers used are listed in Table S1.

### 2.8 Apoptosis analysis

A TUNEL assay was performed to detect apoptosis using a TMR green in situ cell death detection kit (Roche Applied Science), according to the manufacturer's instructions. The fluorescence was detected under a fluorescence microscope at 400× magnification.

### 2.9 Statistical analysis

Data were analysed using Microsoft Excel 2016 and GraphPad Prism 6.0 software (San Diego, CA, USA) and are presented as means ± SEM. The one‐way ANOVA test was performed, and *P* < 0.05 was considered statistically significant.

## 3 RESULTS

### 3.1 Low FGF21 expression is associated with islet β‐cell dysfunction in vivo

**Figure 1:**
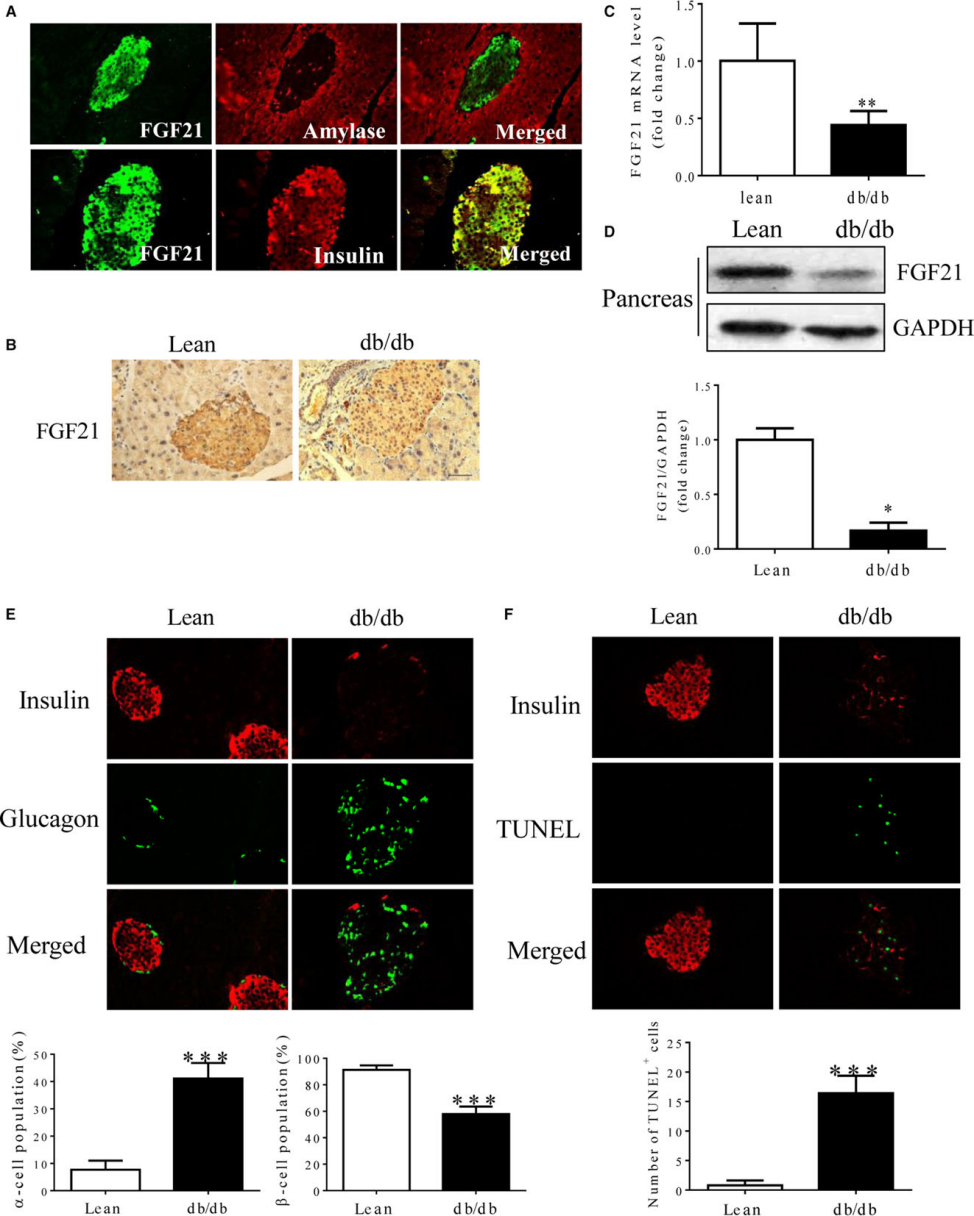
Low FGF21 expression was associated with islet β‐cell dysfunction in db/db mice. (A) Immunofluorescence staining of FGF21 (green), amylase (red) and insulin (red) in pancreatic tissues of 8‐week‐old lean mice. (B) Immunohistochemical staining of FGF21 in pancreatic tissues of 8‐week‐old lean mice and db/db mice. (C) Quantitative PCR analysis of FGF21 mRNA expression. (D) Western blot analysis of FGF21 protein expression, normalized to GAPDH. (E) Immunofluorescence staining of insulin (red) and glucagon (green) in pancreatic sections and quantification of insulin‐ or glucagon‐positive cells. (F) Immunofluorescence staining of insulin (red) and TUNEL (green) staining in pancreatic sections as well as quantification of TUNEL‐positive β‐cells. *P < 0.05, **P < 0.01. Scale bar = 50 μm. (A–C) n = 5. (D) n = 3

To investigate the possible role of FGF21 in maintaining pancreatic islet function, we first examined the expression pattern of FGF21 in pancreatic islets of db/db mice. As shown in Figure 1A and B and Figure S1A, FGF21 was highly expressed in pancreas and specifically expressed in pancreatic islets of lean mice where insulin was produced (Figure 1A) and was down‐regulated in the islets of db/db mice, compared with that in lean controls (Figure 1B). These findings were further confirmed by quantitative PCR and western blot analyses (Figure 1C and D). Notably, however, we found significantly increased circulating FGF21 levels and hepatic FGF21 expression in db/db mice (Figure S1B‐D), consistent with a previous study that plasma FGF21 is mainly liver driving.^27^ In addition, we found that the development of islet β‐cells was impaired in db/db mice, as demonstrated by the decreased population of islet β‐cells specifically expressing insulin and the increased population of islet α‐cells specifically expressing glucagon, compared with the levels in the lean controls (Figure 1E). Consistently, the rate of apoptosis was significantly higher in the islet β‐cells of db/db mice (Figure 1F), compared to that in lean controls. Collectively, these data suggest that pancreatic FGF21 is inhibited in db/db mice, which may be correlated with islet β‐cell dysfunction in T2DM.

### 3.2 Pancreatic FGF21 is essential for islet β‐cell function ex vivo

**Figure 2:**
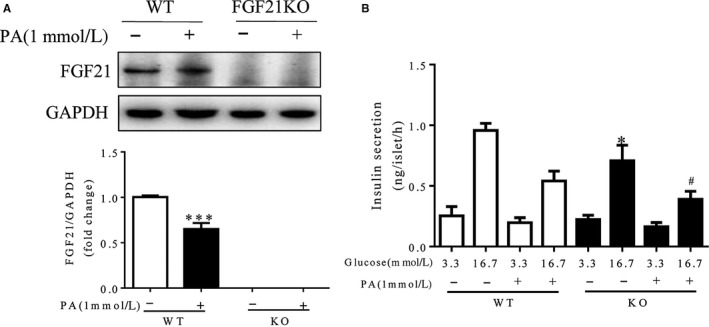
FGF21 deficiency exacerbated palmitate (PA)‐induced islet β‐cell failure. Isolated islets from wild‐type (WT) mice or FGF21 knockout (KO) mice were exposed to 1 mmol/L PA for 48 h followed by (A) western blot assay (***P < 0.001 vs the blank WT group), and (B) the glucose‐stimulated insulin secretion (GSIS) test (*P < 0.05 vs the WT group stimulated with 16.7 mmol/L glucose, ^#^ *P* < 0.05 vs the KO group stimulated with 16.7 mmol/L glucose). (n = 4–5)

As low FGF21 expression in islets may be associated with islet β‐cell dysfunction as described above, we next investigated whether FGF21 plays a role in maintaining islet β‐cell function using a loss‐of‐function assay. As shown in Figure 2A, palmitate induced the failure of cultured wild‐type islets along with the significant down‐regulation of FGF21 in wild‐type islets; whereas FGF21 knockout further exacerbated the adverse effects of palmitate on the islets, as demonstrated by stronger propidium iodide staining in FGF21‐deficient islets than that in wild‐type islets (Figure S2), suggesting the positive role of FGF21 in islets. To further examine the effect of lack of FGF21 on β‐cell defect, we assessed glucose‐stimulated insulin secretion (GSIS) of isolated islets. As expected, there was a marked increase in palmitate‐induced inhibition of GSIS under high‐glucose conditions (16.7 mmol/L) in islets from FGF21 knockout mice, compared with that in islets from wild‐type mice (Figure 2B). Taken together, these results suggest that FGF21 is important for the maintenance of islet β‐cell function.

### 3.3 Overexpression of pancreatic FGF21 improves islet β‐cell function and lipid metabolism disorder in vivo

**Figure 3:**
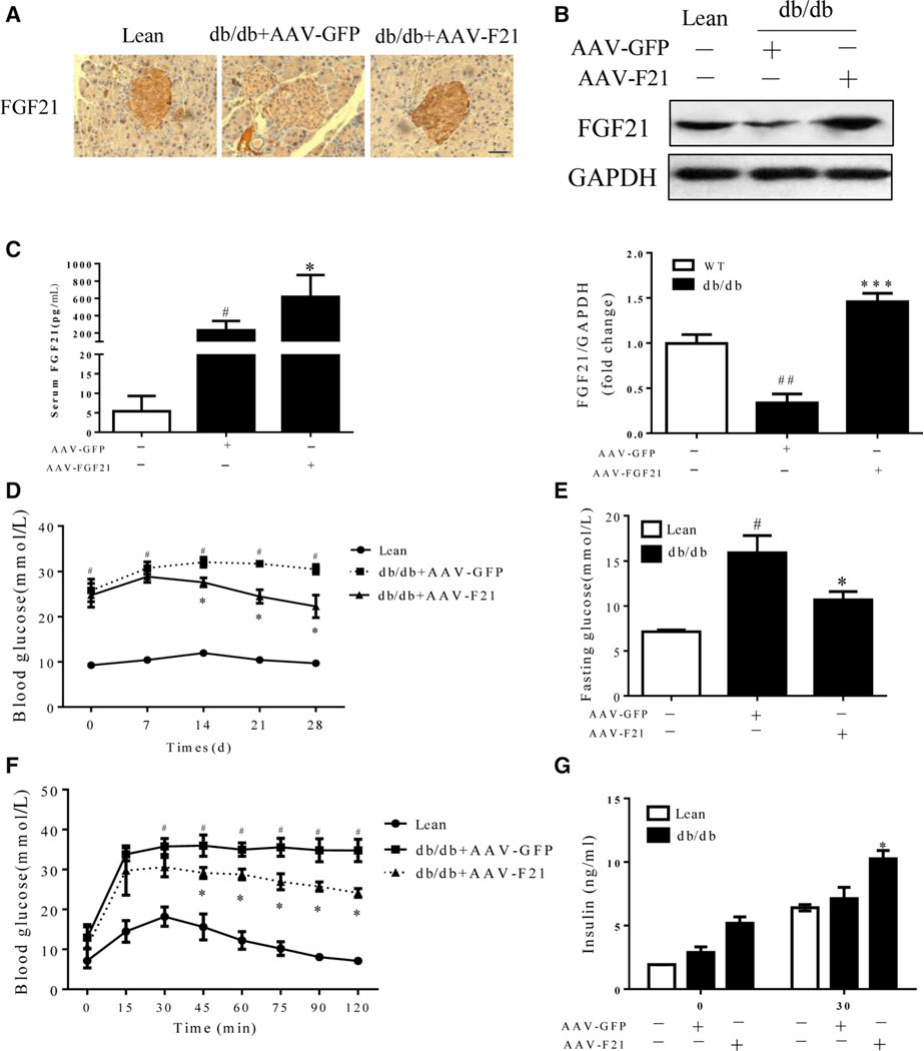
Overexpression of FGF21 ameliorated hyperglycaemia and glucose intolerance in db/db mice. Sixteen‐week‐old db/db mice were intraperitoneally injected with AAV‐FGF21. Serum samples at the indicated time points were collected to determine the blood glucose and circulating FGF21 levels. Age‐matched lean mice with the same genetic background were injected with AAV‐GFP, which served as controls. (A) Immunohistochemical staining of FGF21. (B) Western blot analysis of FGF21 protein expression in islets. (C) Serum FGF21 after 4 weeks of gene delivery in the fed state. (E) Fasting glucose levels after treatment with AAV‐FGF21. (D) Time course of blood glucose levels. (F) GTT results after gene treatment. (G) Plasma insulin levels at 0 and 30 min after intraperitoneal glucose injection were quantified by ELISA. ^#^ *P* < 0.05 vs the lean controls, *P < 0.05 vs db/db mice treated with AAV‐GFP. Scale bar = 50 μm (n = 5)

Overexpression of FGF21 in islets by AAV‐FGF21 was confirmed by immunohistochemistry (Figure 3A). Western blot analysis also showed that the treatments with AAV‐FGF21 increased FGF21 protein expression in islet of db/db mice (Figure 3B). In addition, AAV‐FGF21 further increased plasma FGF21 levels significantly in db/db mice (Figure 3C). The beneficial effect of FGF21 on islet β‐cells was further confirmed by our findings that AAV‐FGF21 treatment attenuated hyperglycaemia (Figure 3D and E) and ameliorated glucose intolerance in db/db mice (Figure 3F and G). However, β‐cell‐specific delivery of FGF21 did not affect the body weights significantly in db/db mice (Figure S3A). The AAV‐FGF21 significantly increased the serum adiponectin and decreased the lipid profiles (Figure S3B‐F) and attenuated fat accumulation both in the liver and pancreas in diabetic mice at weeks 4 after gene treatment (Figure S3G). These data demonstrate that FGF21 plays a protective role against diabetes, which is likely as a result of the improvement of islet β‐cell function by FGF21.

**Figure 4:**
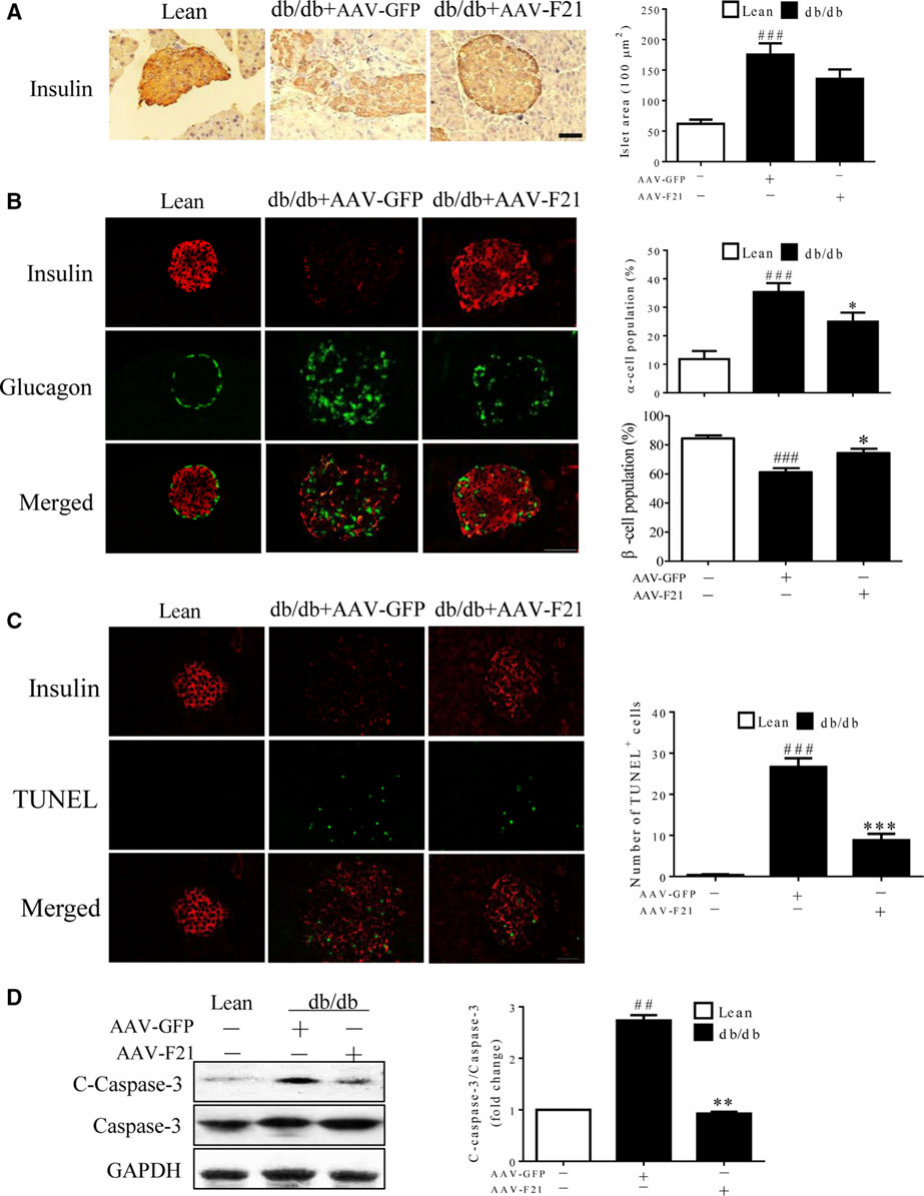
FGF21 improved islet β‐cell function in vivo. Sixteen‐week‐old db/db mice and their lean controls were intraperitoneally injected with AAV‐F21 or AAV‐GFP. Mice were killed at 4 weeks after the AAV injection. (A) Immunohistochemical staining of insulin and measurement of the islet area in pancreatic sections. (B) Immunofluorescence staining of insulin and glucagon in pancreatic sections and quantification of insulin‐ or glucagon‐positive cells. (C) Immunofluorescence staining of insulin and TUNEL in pancreatic sections as well as quantification of TUNEL‐positive β‐cells. (D) Western blot analysis of cleaved and total caspase‐3 expression in the islets isolated from db/db mice, normalized to GAPDH expression. ^###^ *P* < 0.001 vs the lean controls, *P < 0.05, **P < 0.01, and ***P < 0.00 vs db/db mice treated with AAV‐GFP. Scale bar = 50 μm. (A‐C) n = 5. (D) n = 3

To further investigate the role of FGF21 in maintaining islet β‐cell function, a gain‐of‐function assay was performed in db/db mice. The results showed that administration of AAV‐FGF21 effectively promoted insulin production in the islets, improved the distorted islet morphology, and alleviated islet hyperplasia (Figure 4A) as well as increased the islet β‐cell population and decreased the islet α‐cell population in db/db mice (Figure 4B), compared with the AAV‐GFP group. Furthermore, AAV‐F21 also suppressed islet β‐cell apoptosis, as demonstrated by the reduction in both the number of TUNEL‐positive cells and the expression level of cleaved caspase‐3 in the islets of db/db mice (Figure 4C and D). Taken together, these results suggest that pancreatic FGF21 may have a beneficial effect on islet β‐cells in vivo.

### 3.4 FGF21 promotes insulin expression and secretion as well as the expression of insulin gene transcription factors and soluble N‐ethylmaleimide‐sensitive factor activating protein receptor proteins

**Figure 5:**
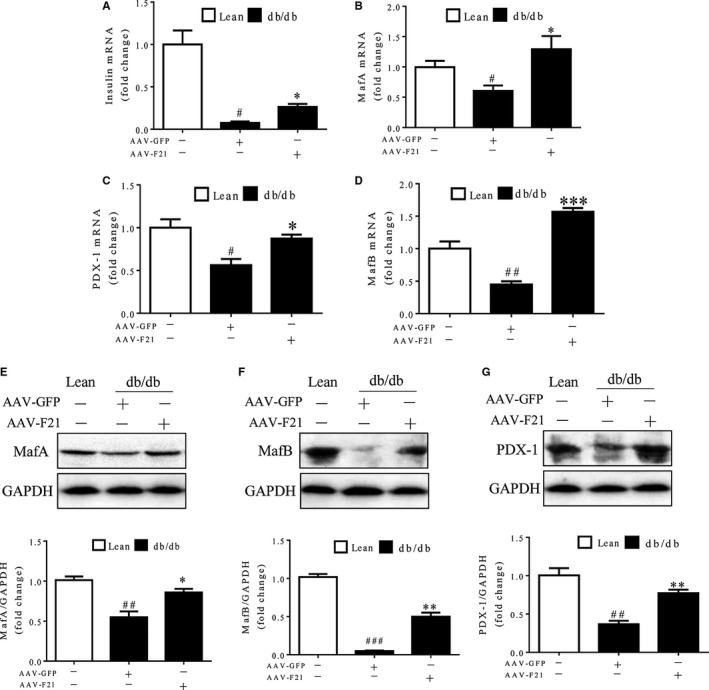
FGF21 promoted insulin secretion along with up‐regulation of insulin gene transcription factors. Sixteen‐week‐old db/db mice and their lean controls were intraperitoneally injected with AAV‐F21 or AAV‐GFP and analysed 4 weeks later. (A–D) Quantitative PCR analysis of insulin, MafA, MafB and PDX1 mRNA expression in the islets. (E–G) Western blot analysis of insulin, MafA, MafB and PDX1 protein expression in the islets. ^#^ *P* < 0.05 and ^##^ *P* < 0.01 vs the lean controls; *P < 0.05, **P < 0.01 and ***P < 0.001 vs db/db mice treated with AAV‐GFP (n = 3‐5 for each group)

To explore the mechanisms underlying the role of FGF21 in islet β‐cells, we investigated the effect of FGF21 on insulin and insulin‐regulating transcription factors like MafA, MafB and PDX‐1 in islets of db/db mice. As shown in Figure 5A‐D, the mRNA expression of these four genes was inhibited in islets of db/db mice, compared with that in islets of lean controls. The inhibited expression was significantly restored by overexpression of FGF21, which was consistent with the protein expression patterns of these four genes (Figure 5E‐G, Figure S4). These data suggest that the protective effect of FGF21 may be attributable to collective up‐regulation of insulin and insulin‐regulating transcription factors.

**Figure 6:**
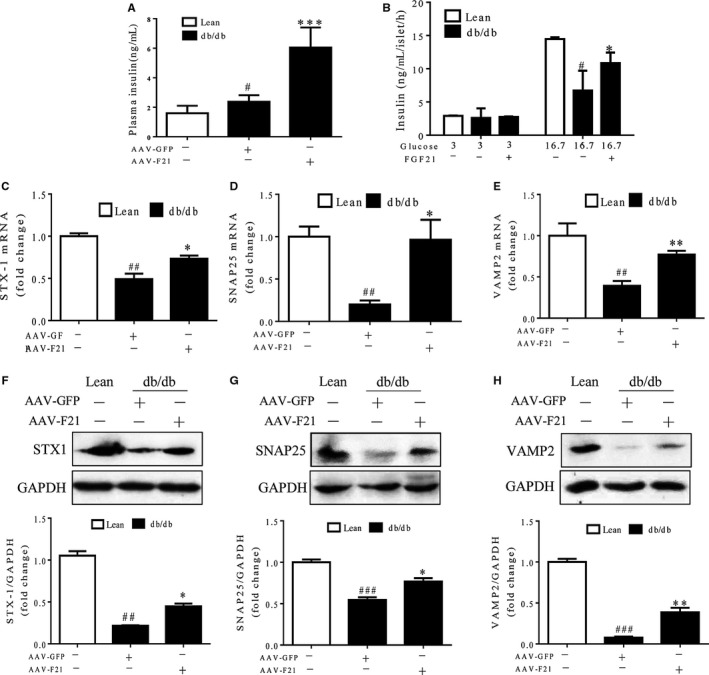
FGF21 promoted insulin secretion along with up‐regulation of SNARE proteins. Sixteen‐week‐old db/db mice and their lean controls were intraperitoneally injected with AAV‐F21 or AAV‐GFP and killed 4 weeks later. (A) The plasma insulin levels were measured immediately following the killing. (B) Static glucose‐stimulated insulin secretion (GSIS) in isolated islets was measured at 1 h after 16.7 mmol/L glucose stimulation. (C–E) Quantitative PCR analysis of STX1, SNAP25 and VAMP2 in isolated islets. (F–H) Western blot analysis of STX1, SNAP25 and VAMP2 in isolated islets. ^#^ *P* < 0.05, ^##^ *P* < 0.01 and ^###^ *P* < 0.001 vs the lean controls; *P < 0.05 and **P < 0.01 vs db/db mice treated with AAV‐GFP. (A) n = 5. (B–H) n = 3‐5

As soluble N‐ethylmaleimide‐sensitive factor attachment protein receptor (SNARE) proteins are the main regulators of insulin secretion,^28^ we next determined whether FGF21 is also involved in the regulation of insulin secretion and insulin secretion‐related SNARE proteins. As shown in Figure 6A and B, both the plasma insulin levels and GSIS were significantly elevated in db/db mice following AAV‐FGF21 treatment. In addition, AAV‐FGF21 treatment restored the mRNA and protein expression of three SNARE proteins (STX‐1, SNAP25 and VAMP2) in db/db mice (Figure 6C‐H, Figure S5). These data suggest that FGF21 may function through promoting insulin secretion and up‐regulating the expression of SNARE proteins in islets.

### 3.5 FGF21 functions in a PI3K/Akt signaling‐dependent manner

**Figure 7:**
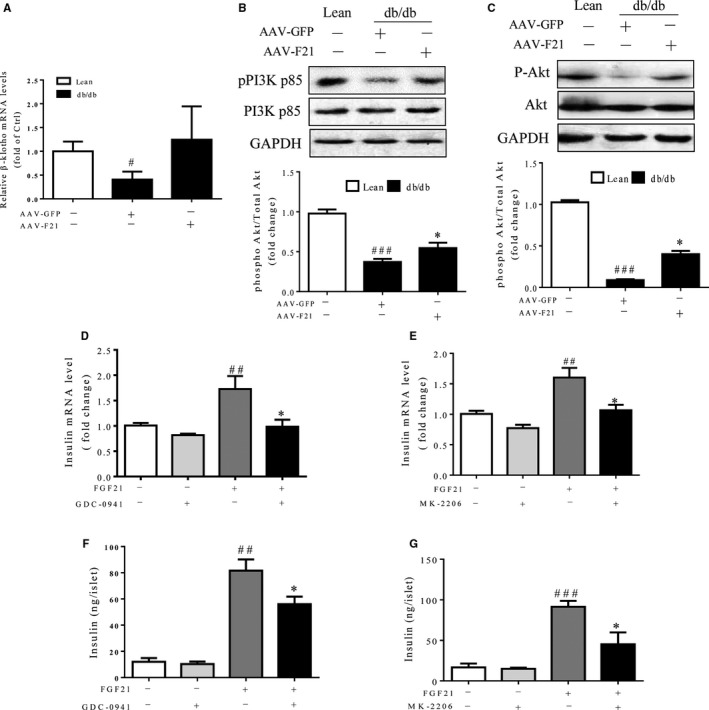
FGF21 regulated insulin expression and secretion in islets via PI3K/Akt signaling pathways. (A) Quantitative PCR analysis of β‐klotho mRNA expression in islet. (B and C) Sixteen‐week‐old db/db mice and their lean controls were intraperitoneally injected with AAV‐F21 or AAV‐GFP and analysed 4 weeks later. Western blot analysis of phosphorylated and total PI3K and Akt protein expression. ^###^ *P* < 0.001 vs the lean controls, *P < 0.05 vs db/db mice treated with AAV‐GFP (n = 3). (D‐G) Islets were isolated from 12‐week‐old lean mice and then treated with 100 ng/mL FGF21 in the absence or presence of PI3K inhibitor (GDC‐0941, 5 μmol/L) or Akt inhibitor (MK‐2206, 1 μmol/L) for 12 h. (D and E) Quantitative PCR analysis of insulin mRNA levels in islets after the treatment. (F and G) Static insulin secretion in isolated islets. ^##^ *P* < 0.01 and ^###^ *P* < 0.001 vs the vehicle group; *P < 0.05 vs the FGF21 group without GDC‐0941 or MK‐2206 (n = 5)

Izumiya et al^29^ proved that FGF21 secreted from muscle is regulated by an Akt1 signaling pathway‐dependent mechanism. In addition, numerous studies have implicated that the PI3K/Akt signaling cascade plays a pivotal role in the regulation of insulin secretion,^30^, ^31^ we sought to examine whether the promotive effects of FGF21 on insulin expression and secretion are dependent on the PI3K/Akt signaling pathway. FGF21 effect results from activation of β‐Klotho. AAV‐FGF21‐induced up‐regulation of β‐klotho in db/db mice was shown Figure 7A. As shown in Figure 7B and C, the activity of PI3K and Akt was significantly suppressed in the islets of db/db mice, compared with lean controls, whereas the suppressive effects were markedly reversed by replenishment of FGF21. Importantly, pharmaceutical inhibition of PI3K/Akt signaling in islets by GDC‐0941 and MK‐2206 significantly inhibited FGF21‐induced insulin expression and secretion in cultured islets (Figure 7D‐G), suggesting that PI3K/Akt signaling is essential for the effects of FGF21 on insulin expression and secretion.

**Figure 8:**
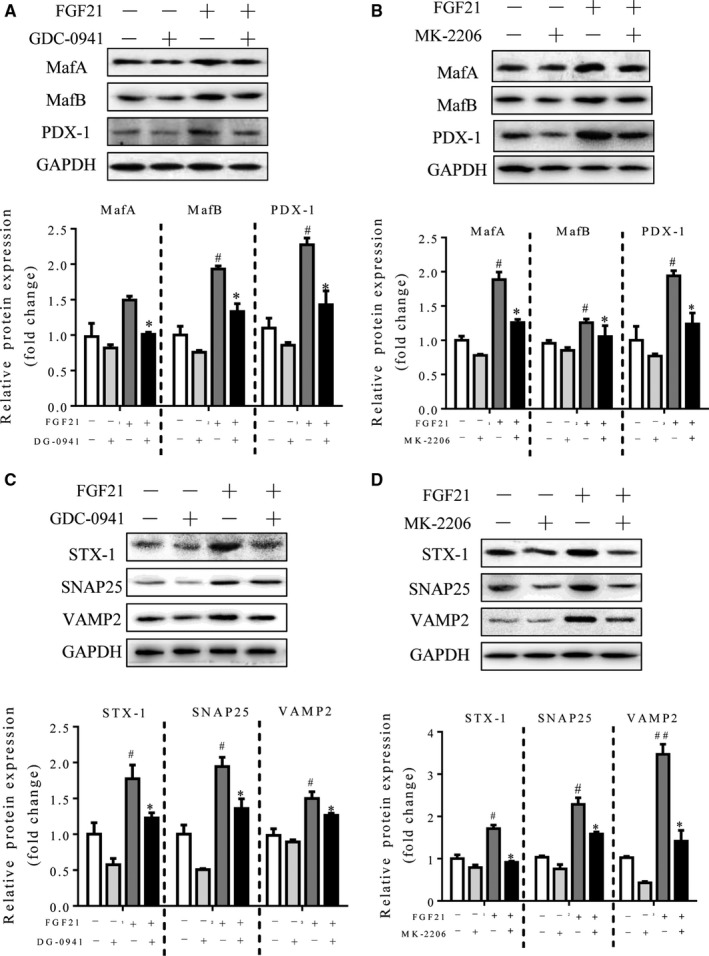
FGF21 regulated insulin gene transcription factors and SNARE proteins via PI3K/Akt signaling pathways. Islets were isolated from 12‐week‐old lean mice and then treated with 100 ng/mL FGF21 in the absence or presence of PI3K inhibitor (GDC‐0941, 5 μmol/L) or Akt inhibitor (MK‐2206, 1 μmol/L) for 12 h. (A and B) Western blot analyses of MafA, MafB and PDX‐1 in islets after the treatment. (C and D) Western blot analyses of SNARE protein expression in islets after the treatment. ^#^ *P* < 0.05 and ^##^ *P* < 0.01 vs the vehicle group; *P < 0.05 vs the FGF21 group without GDC‐0941 or MK‐2206 (n = 4)

Next, we sought to investigate whether FGF21 regulates insulin gene transcription factors and SNARE proteins via PI3K/Akt signaling as well. As shown in Figure 8A‐D, the PI3K/Akt signaling inhibitors GDC‐0941 and MK‐2206 dramatically inhibited the FGF21‐induced expression of insulin gene transcription factors and SNARE proteins. These data suggest that the role of FGF21 in regulating insulin expression and secretion‐related factors is dependent on the PI3K/Akt signaling cascade.

## 4 DISCUSSION

Substantial β‐cell failure occurs at the early stage of T2DM^32^ and serves as a major contributing factor of the pathogenesis of T2DM.^33^ In this study, we demonstrated that FGF21 expression was decreased dramatically in pancreatic islets of db/db mice and that pancreatic islet‐specific overexpression of FGF21 by AAV‐FGF21 significantly improved glycolipid metabolism and alleviated the development of diabetes in db/db mice. On the other hand, our ex vivo studies also indicated that FGF21 deficiency accelerated palmitate‐induced islet injury in cultured islets and attenuated islet function, as demonstrated by the decreased insulin expression and secretion. Collectively, these findings suggest that pancreatic FGF21 is important for promoting insulin expression and secretion, which in turn protect against the progression of diabetes.

Previous studies indicate that FGF21 is a metabolic hormone preferentially expressed in liver tissue with multiple functions.^34^, ^35^ In addition to the liver, FGF21 is highly expressed in adipose tissue and the hypothalamus. Recent studies have shown that FGF21 is also abundant in the pancreas^36^ and plays an anti‐inflammatory role in pancreatitis in mice.^37^ Furthermore, exogenous administration of FGF21 can improve islet transplant success as well as protect islets from glucolipotoxicity and cytokine‐induced apoptosis.^23^, ^25^ A recent study has suggested that FGF21 is highly expressed in exocrine pancreas and functions as an exocrine pancreas secretagogue,^38^ which is not consistent with our findings that the majority of pancreatic FGF21 is synthesized in the islets of C57/BL6 mice at the basal state. Furthermore, another report stated that FGF21^23^ is highly expressed in pancreatic islets, supporting out data, and pancreatic islet and liver may be major sources of systemic FGF21 since chemical ablation of pancreatic β‐cells by streptozotocin and liver‐specific FGF21 KO both result in a dramatic reduction in circulating FGF21.^27^ Our data showed that pancreatic FGF21 expression was extremely weak in db/db mice, along with a decrease in the number of β‐cells as well as islet insulin‐synthesizing and ‐secreting function. Interestingly, hepatic and circulating FGF21 levels were increased in db/db mice along with the decrease in β‐klotho in islets, suggesting that pancreatic β‐cells may have been resistant to hepatic and circulating FGF21 because of the loss of β‐klotho. These findings were consistent with previous works^39^, ^40^ showing that mice lacking β‐klotho are unresponsive to FGF21 stimulation. What is more, the changes of hepatic and pancreatic FGF21 in db/db mice also suggest that the liver is a main source of serum FGF21. In addition, hepatic FGF21 has been found to regulate fatty acid metabolism and improve fatty liver disease in db/db mice.^41^, ^42^ These results suggest that pancreatic FGF21 may be involved in the pathogenesis of diabetes. In support of this observation, AAV‐mediated FGF21 expression in islets significantly decreased the glucose levels and improved glucose tolerance in db/db mice.

It is well established that insulin expression is mediated by MafA, MafB and PDX‐1^43^ and that insulin secretion is mediated by SNARE proteins in β‐cells.^28^ Under diabetic conditions, the expression of MafA, MafB and PDX‐1 is markedly decreased in β‐cells, leading to suppressed insulin biosynthesis and secretion. Meanwhile, the secretory vesicle (v) SNAREs (VAMP2) interact with the target membrane (t) SNARE proteins (STX‐1 and SNAP25) to form a stable heterotrimeric complex to facilitate membrane fusion,^44^, ^45^, ^46^ and their deficiency is responsible, in part, for the impaired insulin secretion in both human T2DM and animal models of T2DM.^5^, ^47^ Consistent with these findings, SNARE proteins as well as MafA, MafB and PDX‐1 were found to be significantly decreased in db/db mice. Importantly, AAV‐mediated β‐cell‐specific overexpression of FGF21 significantly increased the mRNA and protein expression levels of MafA, MafB and PDX‐1 as well as SNARE proteins in the islets of mice. Functionally, overexpression of FGF21 attenuated hyperglycaemia and insulin storage, improved glucose tolerance and preserved islet β‐cells from apoptosis. Consistently, our data also indicate that insulin was significantly up‐regulated along with increased GSIS in isolated islets from db/db mice after AAV‐FGF21 treatment. These results suggest that FGF21 may protect against T2DM in mice through promoting insulin expression and secretion as a result of collective up‐regulation of MafA, MafB and PDX‐1 as well as SNARE proteins in islets of db/db mice.

Growing evidence has shown that PI3K/Akt signaling plays a critical role in the regulation of β‐cell function^30^, ^48^ and that inactivation of PI3K/Akt signaling in β‐cells leads to glucose intolerance and decreases insulin secretion in response to glucose challenge as a result of down‐regulation of SNARE complex proteins.^48^ Consistently, we demonstrated for the first time that PI3K/Akt signaling was significantly inhibited in the islets of db/db mice, whereas AAV‐FGF21 significantly promoted the phosphorylation of PI3K and Akt, in parallel with an increase in insulin expression and a decrease in β‐cell apoptosis. On the other hand, our ex vivo data also indicated that treatment with FGF21 protein also increased insulin expression and activated PI3K/Akt signaling in cultured islets. However, this effect was partially reversed in the presence of PI3K/Akt signaling inhibitors, indicating that other pathways may be involved in the FGF21‐mediated signal transduction. We also found that mTOR is hyperactivated in diabetic islets and FGF21 up‐regulation inhibits mTOR activity while promoting insulin secretion in db/db mouse islets (data not shown). Taken together, these data suggest that the beneficial effects of FGF21 on insulin synthesis and secretion may be at least partially attributed to activation of PI3K/Akt signaling.

In summary, our findings demonstrate that pancreatic FGF21 protects β‐cells from T2DM‐induced injury through promoting insulin synthesis and secretion, which are mediated by PI3K/Akt signaling‐dependent up‐regulation of MafA, MafB and PDX‐1 as well as SNARE proteins. In future studies, the protective role of pancreatic FGF21 in T2DM needs to be further confirmed using a β‐cell‐specific knockout FGF21 mouse model.

## CONFLICT OF INTEREST

All authors declared that there was no conflict of interests involved in this study.

## AUTHOR CONTRIBUTION

This study was conceived and designed by Zhuofeng Lin and Xiaokun Li and supervised by Xuebo Pan, Xiaokun Li and Zhuofeng Lin. Yingying Pan contributed to animal model establishment, experimentation, data collection, data analysis, data interpretation, literature searching, figure generation and manuscript writing. Baile Wang contributed to the experimental design, data interpretation, figure generation and manuscript editing. Rongrong Xiong, Zhichao Fan, Jujia Zheng, Yanna Ye, Saisai Zhang and Qinyao Li carried out the experiments. Chaoming Wu and Fanghua Gong provided relevant information. All authors listed on the manuscript agreed to the manuscript submission.
