# Supplementary figures and images for: Pancreatic fibroblast growth factor 21 protects against type 2 diabetes in mice by promoting insulin expression and secretion in a PI3K/Akt signaling‐dependent manner
Source: J Cell Mol Med. 2018 Nov 20;23(2):1059–71. doi: 10.1111/jcmm.14007 (PMC6349243; doi:10.1111/jcmm.14007)

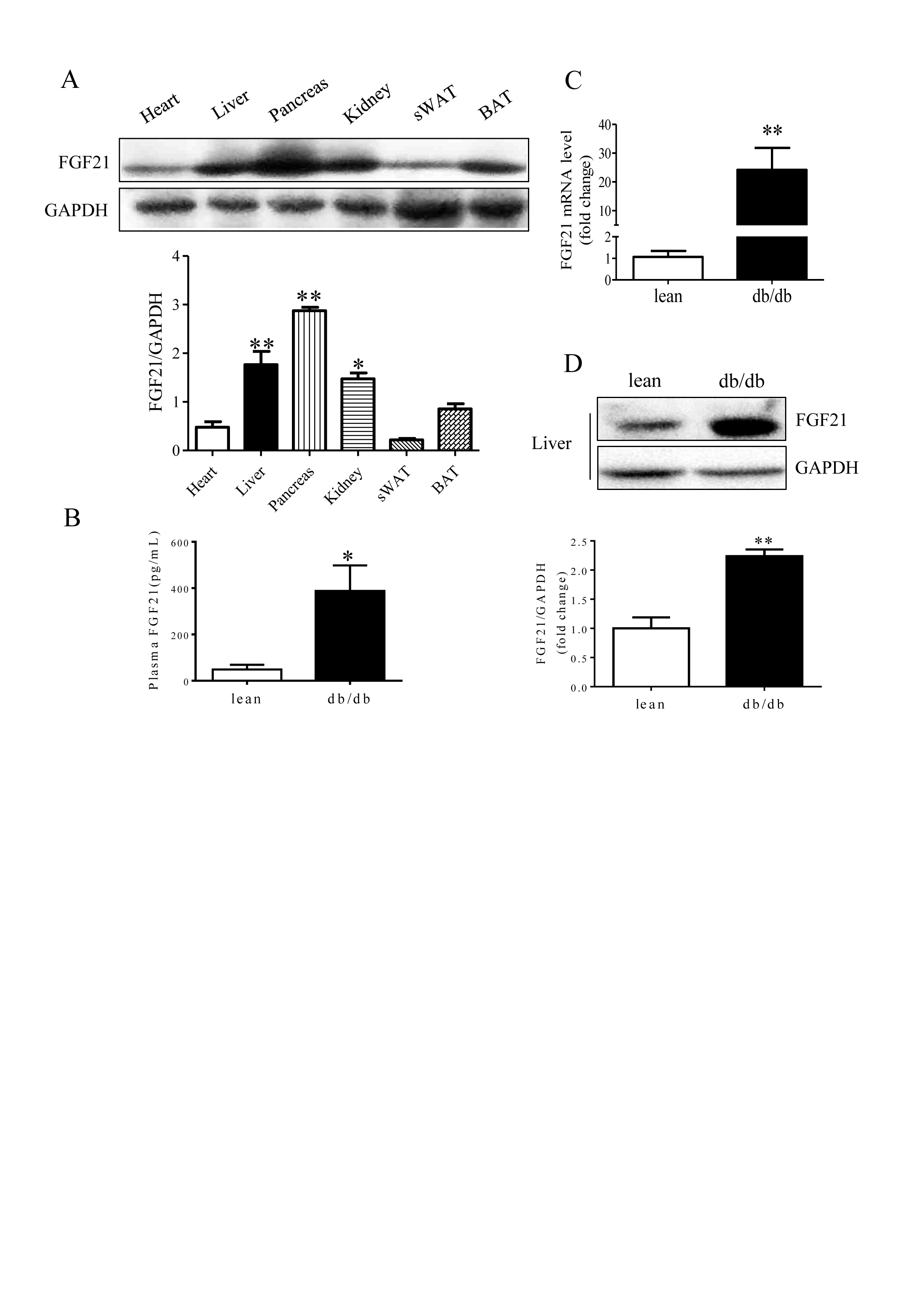

Supplement: Supplementary file 1 [file JCMM-23-1059-s001.tif]

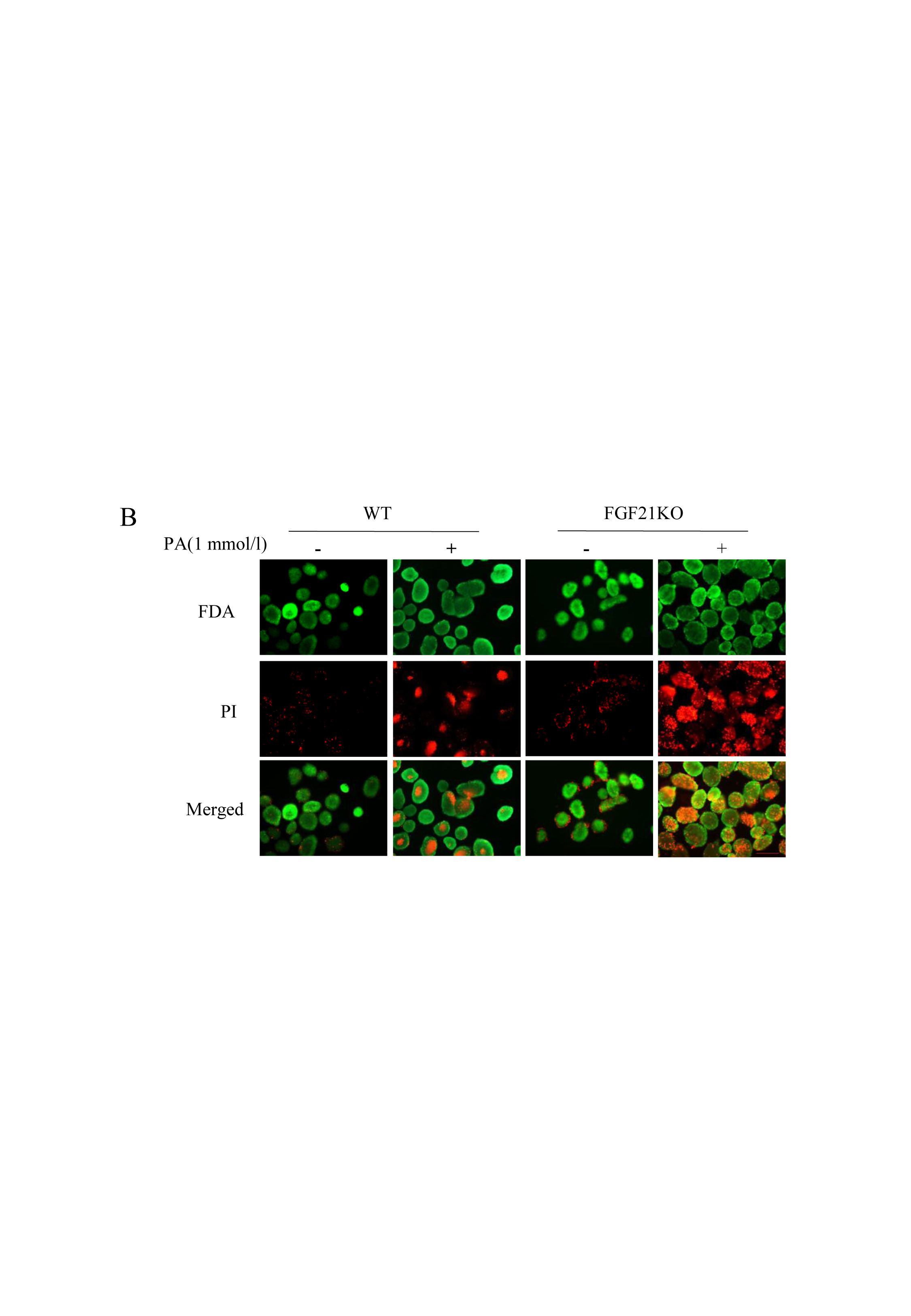

Supplement: Supplementary file 2 [file JCMM-23-1059-s002.tif]

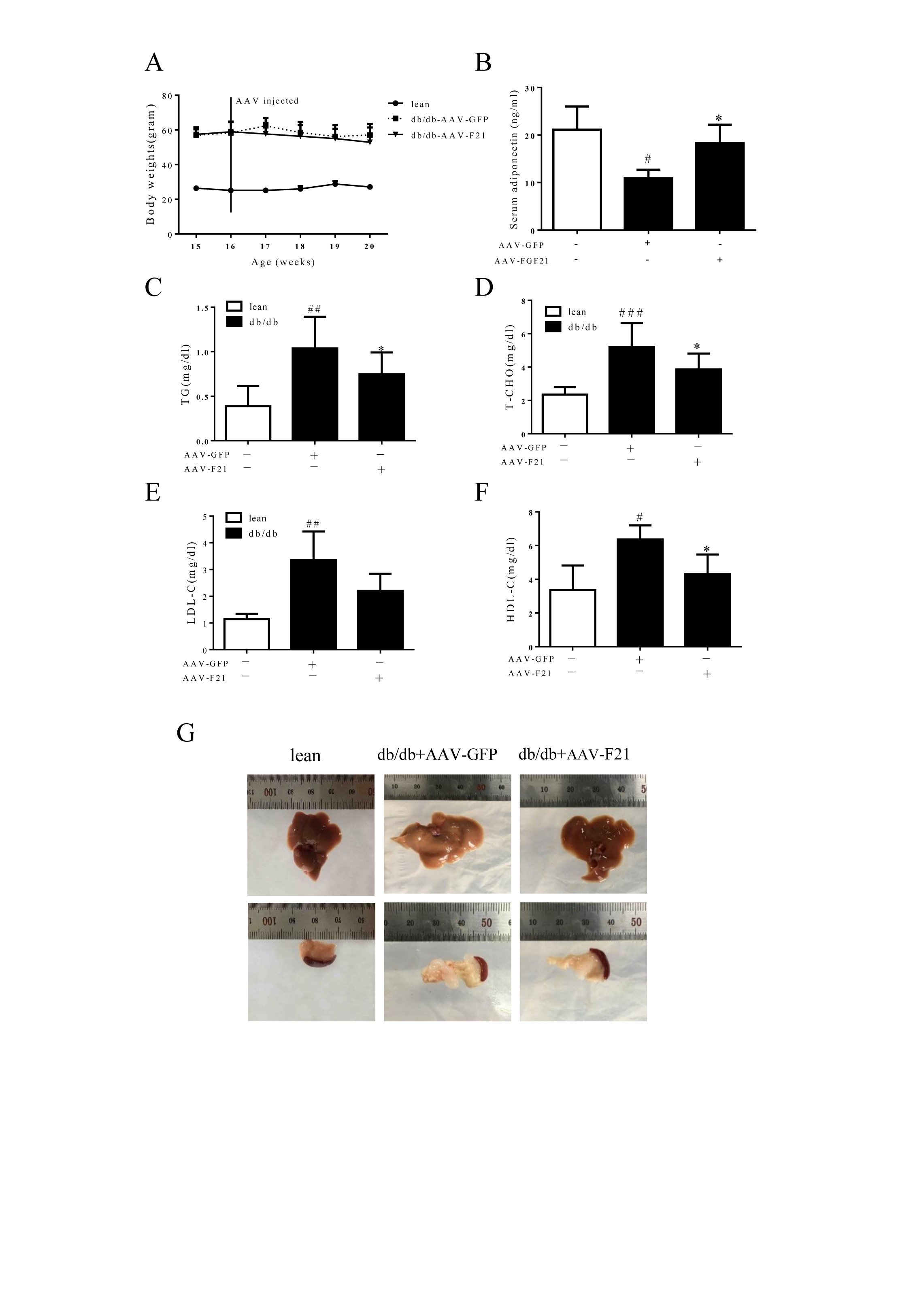

Supplement: Supplementary file 3 [file JCMM-23-1059-s003.tif]

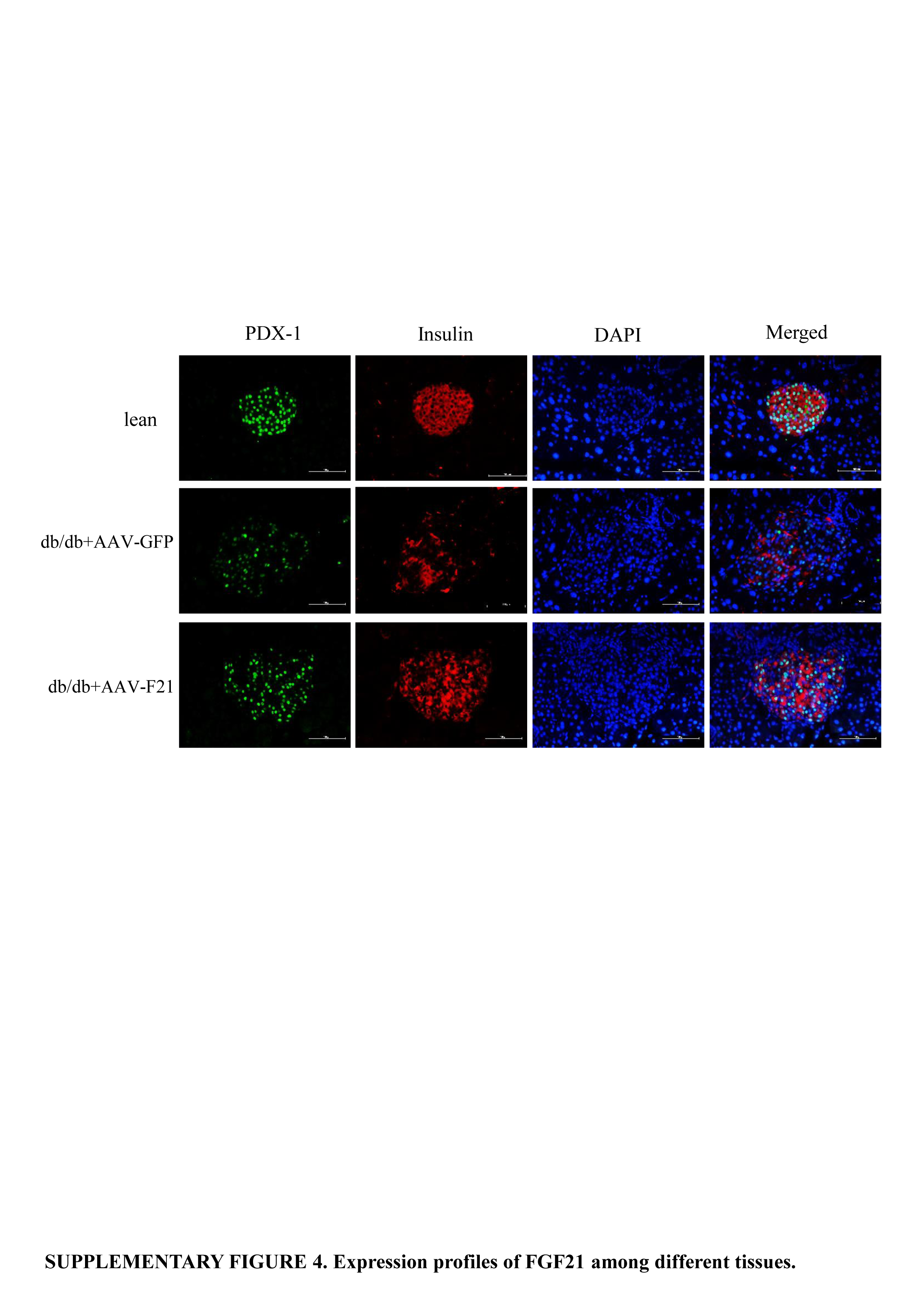

Supplement: Supplementary file 4 [file JCMM-23-1059-s004.tif]

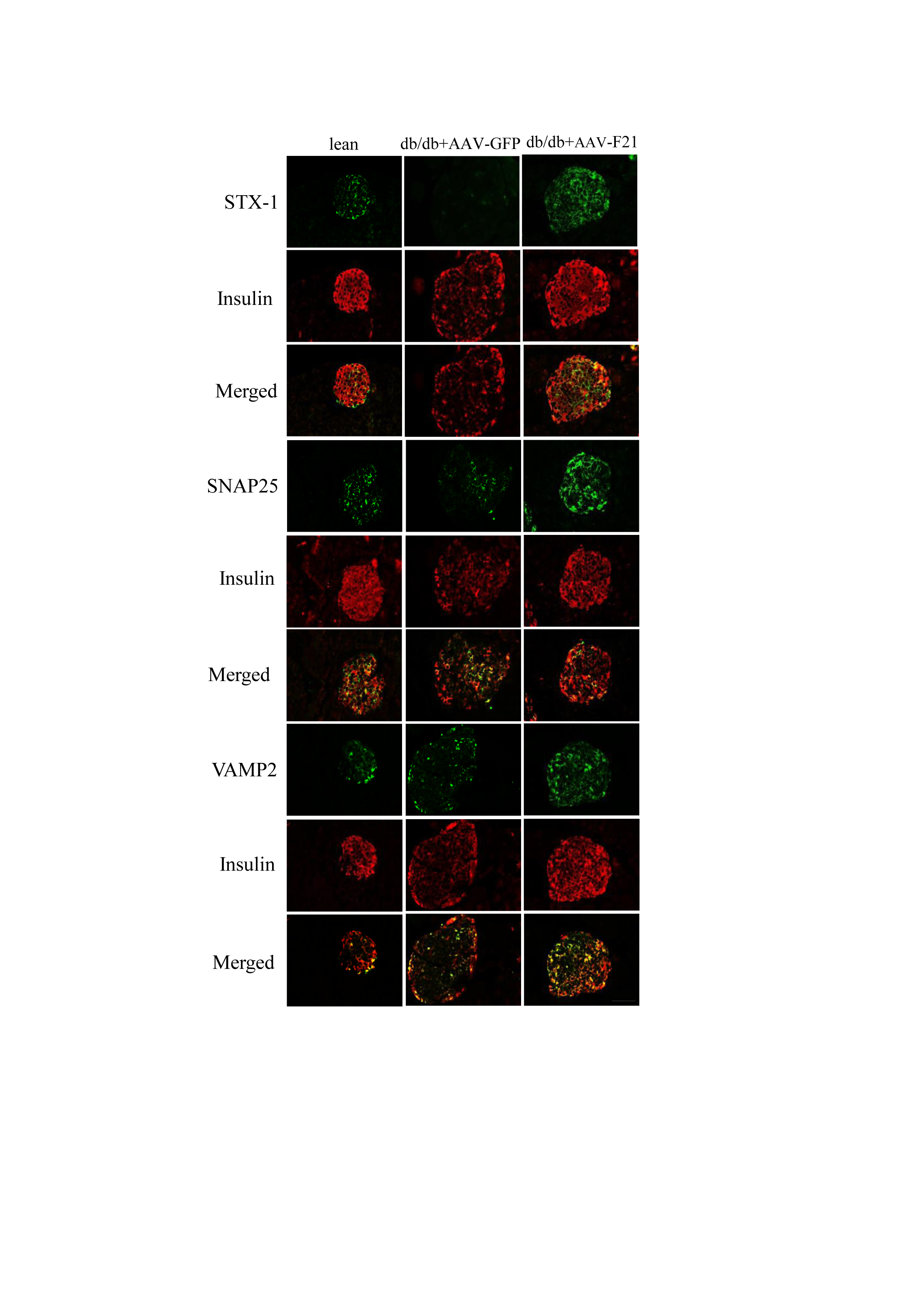

Supplement: Supplementary file 5 [file JCMM-23-1059-s005.tif]
