# Supplementary material for: Pancreatic fibroblast growth factor 21 protects against type 2 diabetes in mice by promoting insulin expression and secretion in a PI3K/Akt signaling‐dependent manner
Source: J Cell Mol Med. 2018 Nov 20;23(2):1059–71. doi: 10.1111/jcmm.14007 (PMC6349243; doi:10.1111/jcmm.14007)
Supplement: Supplementary file 6 [file JCMM-23-1059-s006.docx]

**Supplementary Table 1** Primer sequences used for real-time PCR analysis

| **Genes** | **Forward** | **Reverse** |
| --- | --- | --- |
| FGF21 | 5＇GCCTTGAAGCCGGGAGTTATT 3＇ | 5＇GTGGAGCGATCCATACAGGG 3＇ |
| Insulin  β-klotho | 5＇AGCGTGGCTTCTTCTACACAC 3＇  5＇ACGACCCGACGAGGGCTGTT 3＇ | 5＇CTGGTGCAGCACTGATCTACA 3＇  5＇GGAGGAGACCGTAAACTCGGGCTTA 3＇ |
| SNAP25 | 5＇ GTGAGGAATTGGAAGACATGC 3＇ | 5＇GCCTTGCTCTGGTACTTGACG 3＇ |
| VAMP2 | 5＇CCCACACACCAGGTTTTCTGT 3＇ | 5＇GCAGGGGACACTGGGATAATA 3＇ |
| STX-1 | 5＇ATGGAGAAGGCTGATTCCAAC 3＇ | 5＇ CCATGAGAGAAGCATGAAGGA 3＇ |
| PDX-1 | 5＇GGTATAGCCGGAGAGATGC 3＇ | 5＇CTGGTCCGTATTGGAACG 3＇ |
| MafA | 5＇AGGAGGAGGTCATCCGACTG 3＇ | 5＇CTTCTCGCTCTCCAGAATGTG 3＇ |
| MafB | 5＇TTCGACCTTCTCAAGTTCGACG 3＇ | 5＇TCGAGATGGGTCTTCGGTTCA 3＇ |
| GAPDH | 5＇GCACAGTCAAGGCCGAGAAT 3＇ | 5＇GCCTTCTCCATGGTGGTGAA 3＇ |
